# Adipocytokine Associations with Insulin Resistance in British South Asians

**DOI:** 10.1155/2013/561016

**Published:** 2013-02-25

**Authors:** D. R. Webb, K. Khunti, S. Chatterjee, J. Jarvis, M. J. Davies

**Affiliations:** ^1^Department of Cardiovascular Sciences, University of Leicester, Leicester LE3 9QP, UK; ^2^Leicester Diabetes Center, Leicester General Hospital, Ward 5 (Broadleaf), Gwendolen Road, Leicester LE5 4PW, UK; ^3^Department of Health Sciences, University of Leicester, Leicester LE1 6TP, UK; ^4^Buckinghamshire Hospitals NHS Trust, Buckinghamshire HP7 OJD, UK

## Abstract

*Aims*. Adipocytokines are implicated in the pathogenesis of type 2 diabetes and may represent identifiable precursors of metabolic disease within high-risk groups. We investigated adiponectin, leptin, and TNF-**α** and assessed the contribution of these molecules to insulin resistance in south Asians. *Hypothesis*. South Asians have adverse adipocytokine profiles which associate with an HOMA-derived insulin resistance phenotype. *Methods*. We measured adipocytokine concentrations in south Asians with newly diagnosed impaired glucose tolerance or Type 2 Diabetes Mellitus in a case-control study. 158 (48.5% males) volunteers aged 25–75 years with risk factors for diabetes but no known vascular or metabolic disease provided serum samples for ELISA and bioplex assays. *Results*. Total adiponectin concentration progressively decreased across the glucose spectrum in both sexes. A reciprocal trend in leptin concentration was observed only in south Asian men. Adiponectin but not leptin independently associated with HOMA-derived insulin resistance after logistic multivariate regression. *Conclusion*. Diasporic south Asian populations have an adverse adipocytokine profile which deteriorates further with glucose dysregulation. Insulin resistance is inversely associated with adiponectin independent of BMI and waist circumference in south Asians, implying that adipocytokine interplay contributes to the pathogenesis of metabolic disease in this group.

## 1. Introduction

The role of obesity in the pathogenesis of metabolic disease has received considerable attention since the discovery of biologically active adipose-tissue-derived circulatory proteins (adipocytokines) [1–3]. It is proposed over- or underproduction of adipocytokines in overweight individuals generates an adipose-specific inflammatory response which may be an important determinant of Type 2 Diabetes Mellitus (T2DM) [4–6]. Prospective evidence exists for this within certain populations, with low concentrations of the adipocytokine adiponectin correlating strongly with insulin resistance syndromes and or incident T2DM independent of obesity [4–15]. Adiponectin may decrease T2DM risk via a number of mechanisms including hepatic fatty acid oxidation, enhanced peripheral glucose uptake, and stimulated insulin secretion [16]. 

The identification of particular biomarker profiles within well-defined high-risk groups may therefore provide important pathogenic insight as well as potential clinical utility through predictive capacity [17]. Of particular interest are United Kingdom populations tracing first or second ancestry to the Indian subcontinent. Collectively termed South Asians, it appears this diaspora is particularly susceptible to the effects of Western urbanisation and manifest a disproportionate prevalence of premature T2DM [18]. There is some evidence that South Asians have lower adiponectin and higher circulating hsCRP concentrations than White Europeans even in the absence of BMI-defined obesity and glucose dysregulation. This implies hypoadiponectinemia may be a generalised phenomenon within this group and a possible mediator of premature metabolic disease [4, 19–23]. The majority of biomarker studies to date have attempted to correlate various topographical measurements and markers of metabolic dysfunction with adiponectin [24–28]. We are unaware of any previous studies categorising adipokines across the glucose spectrum in UK South Asians and then establishing their relationship with insulin resistance independent of measures of obesity. 

This study aimed to firstly characterise a range of adipokines (adiponectin, leptin, Tumour Necrosis Factor-*α* (TNF-*α*)) within age-gender-matched groups of South asians with normal glucose tolerance (NGT), impaired glucose tolerance (IGT) and Type 2 Diabetes mellitus (T2DM) and secondly determine adipocytokine interactions with known predictors of metabolic risk and insulin resistance within this group. 

## 2. Methods

### 2.1. Subjects

**Figure 1:**
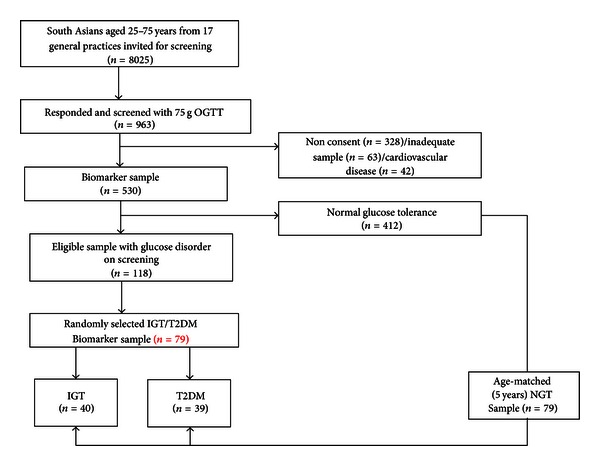
Recruitment and sampling procedure flow diagram.

This was a retrospective case-control study embedded within a population-based screening programme for diabetes. Seventeen general practices initially invited listed patients with at least one risk factor for diabetes to a hospital or community-based screening appointment [29]. The response rate amongst invited south Asian subjects was 12%. Nine hundred and sixty-three male and female volunteers aged 25–70 underwent a standard 75 g oral glucose tolerance test (OGTT). A diagnosis of normal glucose tolerance (NGT), impaired glucose tolerance (IGT), or Type 2 Diabetes (T2DM) was made using 1999 WHO criteria [30]. Additional consent was obtained for adipocyte biomarker analyses in south Asian individuals with no history of cardiovascular disease (Figure 1). Approval was granted by the local research and ethics committee as a substudy amendment to the original screening programme protocol. The investigation was conducted in accordance with the principles outlined in the Declaration of Helsinki.

### 2.2. Recruitment and Patient Flow

Of the volunteers reporting south Asian ethnicity in the parent screening study, 530 were eligible for and consented to the temporary storage of their serum for future adipocytokine biomarker analysis. Twenty-two percent of this group had a WHO-defined glucose disorder and their samples were selected within respective T2DM and IGT categories to produce equal sample sizes across the glucose spectrum. All T2DM cases and 50% (40/79) of IGT subjects were selected for biomarker analysis and an age-sex-matched normal glucose tolerant control allocated by an independent researcher blinded to biomarker data. There were no statistically significant demographic differences between those consenting for (*n* = 530) and those meeting the inclusion criteria (no cardiovascular disease) but not consenting for biomarker analysis (*n* = 328) (Figure 1). 

### 2.3. Biomedical Measurements

Baseline demographic data captured at screening included age, sex, smoking behaviour, body mass index (BMI), waist circumference, and self-reported history of cardiovascular disease or its treatment. Ethnicity status was self-assigned using UK population census categories. Weight was measured to the nearest 0.1 kg using standard weighing scales. Height was measured to the nearest 0.1 cm using a stadiometer. Waist circumference was measured by trained staff using a nonstretching measuring tape over the tops of the iliac crests [31]. Hip circumference was measured over the greatest protrusion of the gluteal muscles. Blood pressure was measured according to a standardised operating procedure using a calibrated sphygmomanometer and brachial inflation cuff (HEM-7200 M3, Omron Healthcare, Kyoto, Japan). 

### 2.4. Biomarker Measurements

Biomarker and insulin samples were handled separately to screening measurements. They were immediately centrifuged and stored at −80°C in 200 *μ*L aliquots to minimise repeated defrosting cycles. These analyses were performed in a single University research laboratory with expertise in adipocytokine assays. Adiponectin, leptin, and TNF-*α* were all analysed using bioplex assay according to the manufacturer's instructions (Linco Research Inc., St. Charles, MO, USA). Bioplex assay sample measurement using fluorescent microbead technology allowed simultaneous quantitation of several target proteins within a single serum sample of 50–100 *μ*L. For all the assays, the filter plate was prewetted using a specific wash buffer and then 65 *μ*L of assay buffer followed by 10 *μ*L of calibrator, controls or 10 *μ*L of diluted serum samples were added to the appropriate wells. The bead bottle was thoroughly vortexed for 1 minute before adding 25 *μ*L of the bead suspension to each well, taking care to mix intermittently to avoid settling. The filter plate was sealed, covered with aluminium foil, and incubated for 16–18 hours overnight at 2–8°C. The fluid was then gently removed by vacuum extraction and the plate was washed three times with wash buffer. Fifty microlitres of detection antibody cocktail was added to each well and then the plate was resealed, covered, and incubated with agitation for 30 minutes at room temperature. After removing contents by vacuum, the plate was washed three times, and then 50 *μ*L of streptavidin-phycoerythrin was added to each well. After further identical incubation at 30 minutes, the contents were removed by vacuum, the plate was washed three times and 100 *μ*L of sheath fluid was added to all wells. The beads were resuspended by shaking on a plate shaker for 5 minutes before the plate was run on the analyser. For bioplex, intraassay coefficient of variation was 1.4–7.9% and inter-assay coefficient of variation was <15%. 

### 2.5. Statistical Analysis

A power calculation was undertaken for a biomarker (adiponectin) comparison based upon available data in south Asian subjects with type 2 diabetes [24]. A total sample size of 34 would give 80% power at the 5% level to detect a one standard deviation difference between south Asian IGT and T2DM groups. All values are given as mean ± standard deviation (SD) unless otherwise stated. Skewed distributions were log transformed. *T*-tests or non parametric comparisons of median values were used to determine statistical differences in biomedical data across glucose categories. HOMA-derived insulin resistance (HOMA-IR) was calculated as fasting insulin (*μ*Uml^−1^)  × fasting glucose (mmoll^−1^)/22.5, with a set value of more than 3.0 indicating likely significant Insulin resistance. Conditional logistic regression analysis following logarithmic transformation of selected variables was used to determine independent biomarker relationships with insulin resistance. Only variables considered to be of importance and those significant at the level of bivariate analysis (see Supplementary Material available at http://dx.doi.org/10.1155/2013/561016) were entered simultaneously into the model. Categorical variables were coded as follows: gender: male or female, smoking: active or inactive, cardiovascular disease or lipid lowering medication: active prescription or no prescription. Summary measures of goodness of fit were performed ahead of regression analyses. Significance was assumed if *P* < 0.05. All statistical analyses were carried out using SPSS statistical software version 20.0 (SPSS, Chicago, IL, USA).

## 3. Results

158 Indian south Asian subjects were included, 79 with normal glucose tolerance (NGT), 40 with impaired glucose tolerance (IGT) and 39 with Type 2 Diabetes (T2DM). The mean age of the study population was 53.6 years with men and women equally represented. Biomedical characteristics categorised by glucose status are displayed in Table 1. There were statistically significant differences in Body Mass Index (BMI), waist circumference, waist hip ratio (WHR), serum triglycerides, glucose indices (fasting and two-hour plasma glucose), and derived insulin resistance (HOMA-IR) between NGT and T2DM categories. Significant differences were also observed between IGT and T2DM groups for these parameters and serum HDL-cholesterol. Fewer NGT controls were prescribed antihypertensive and or lipid lowering therapies than either of the IGT or T2DM comparator groups. 

Table 2 depicts mean biomarker concentrations across NGT, IGT, and T2DM glucose categories. Adiponectin concentration decreased and leptin concentration increased across the glucose spectrum. An incremental reduction in adiponectin between NGT and T2DM groups reached statistical significance in women. Conversely a statistically significant increase in leptin concentration between NGT and T2DM was observed in men. There were no statistically significant differences in TNF-*α* concentration across glucose categories.

Table 3 demonstrates the independent effects of adiponectin, leptin, and TNF-*α* on insulin resistance (defined by HOMA-IR > 3.0) using conditional logistic regression. Omnibus test of model coefficients indicated a good fit for all models (e.g., for adiponectin chi [2] 64.4 *P* < 0.001). An independent association was observed between the defined insulin resistant state and adiponectin in models adjusting for the effects of age, gender, BMI, waist circumference, smoking, cardioprotective medications, and lipids. No independent associations of insulin resistance with either Leptin or TNF-*α* were observed in this selected south Asian group in the two models adjusting for these parameters.

## 4. Discussion

This study adds to the body of evidence implicating adipocytokine activity (or inactivity) in the pathogenesis of metabolic disease. South Asians, whether “westernised” or residing on the Indian subcontinent appear to have an adverse adipocytokine profile characterised by low total or fractionated hexameric adiponectin and increased leptin. Here we demonstrate an independent association of adiponectin with insulin resistance in a group known to develop diabetes and coronary heart disease earlier than indigenous white European populations.

Like others, we found that adiponectin is low in south Asians compared with similar published data for white Europeans and other races [23–28]. We found an independent relationship between HOMA measured insulin resistance and total adiponectin in south Asians after adjusting for the effects of age, BMI, waist circumference, cardiovascular protective medication, and lipids. This was an expected finding as plasma adiponectin has previously been shown to predict type 2 diabetes within high-risk Asian, Native American and Caucasian populations [32]. Cross-sectional studies examining glucose and adiponectin relationships have demonstrated either strong relationships [19, 25, 33] with HOMA or independent relationships confined to various glucose indices [23–25]. A recent study by Luo et al. demonstrated that total adiponectin associates with IGT and T2DM in Asian Indian women [26]. Low adiponectin levels in pregnancy have recently been shown to predict postpartum insulin resistance and beta-cell dysfunction^,^indicating a major role in the pathogenesis of T2DM [34]. Interestingly, weight loss (and increased insulin sensitivity) through calorie restriction does not appear to effect presumed hyperinsulinaemia-induced reduction in adiponectin in obese women, suggesting pre-determined genetic causality or alternative adipocytokine biological activity [35]. We found little evidence of this on our selected population with no independent association of insulin resistance with either serum leptin or TNF-*α*. Adiponectin-insulin resistance interplay appears more complex than the relatively simplistic paradigm of causality currently postulated. Alternative hypotheses are emerging in which insulin sensitivity may mediate adiponectin processing and release [36]. Longitudinal work is warranted to further investigate this relationship in South Asians and to characterise primary genetic alterations that may predispose this group to hypoadiponectinemia.

Leptin is an adipocytokine with a key role in the hypothalamic satiety response regulating energy metabolism. Some studies in south Asians link this molecule to inflammation, insulin resistance and other cardiovascular risk factors [37, 38]. TNF-*α* is markedly upregulated in obese states and probably promotes insulin resistance by interfering with insulin receptor signalling. TNF-*α* concentrations are higher in urban compared with rural South Asians living in India [39]. Lack of any correlation between leptin/TNF-*α* and insulin resistance in our south Asian population was an unexpected finding. The absence of an association between insulin resistance and leptin independent of BMI or waist circumference in this high risk group suggests this molecule may not be a key driver to hyperinsulinemia in the advanced stages of dysmetabolism.

 We recognize a number of limitations to our study design. Firstly, power to detect differences in biomarker concentrations other than adiponectin may be insufficient, especially for subgroup and gender comparisons. Secondly, the cross-sectional design of the study cannot infer causality. Longitudinal studies with many years of followup are required to determine true relationships between biomarker profiles and metabolic disease/diabetes risk. Thirdly, there is some evidence that adiponectin subfractions (high molecular weight adiponectin—12–18 multimers) rather than the total concentration measured here provides a better measure of biological activity and is a strong predictor of diabetes [40]. This could provide an explanation for the lack of independent correlation of adiponectin with insulin resistance in other south Asian groups although a recent prospective study showed similar associations of total and high molecular weight adiponectin with incident diabetes [32]. There remain relatively few studies in migrant south Asian populations who are at high risk of T2DM and strengths of this approach included the robust phenotyping methodology employed and cardiovascular disease free sample.

In summary, we have found an independent association of the biomarker Adiponectin with insulin resistance in South Asians. Further work to elucidate exactly how adipocytokine interplay contributes to metabolic risk across ethnic groups is needed.

## Supplementary Material

Bivariate associations with HOMA-IR derived insulin resistance for the entire study population are provided as supplementary electronic material.

## Figures and Tables

**Table 1:** Biomedical characteristics of the study population defined by normal glucose tolerance (NGT), impaired glucose tolerance (IGT), and newly diagnosed type 2 Diabetes (T2DM).

	NGT	IGT	T2DM	*P * NGT versus T2DM	*P * IGT versus T2DM
*n *	79	40	39		
Age (years)	52.1 ± 9.8	55.1 ± 11.7	52.7 ± 9.2	0.08	0.18
Male sex	40 (50)	22 (54)	17 (44)	0.37	0.96
Active Smoking	8 (10)	6 (15)	6 (15)	0.71	0.85
BMI (kgm^−2^)	26.6 ± 4.2	29.1 ± 4.9	30.1 ± 5.8	0.01	0.26
Waist circumference (cm)	92.7 ± 10.6	96.9 ± 10.1	100.8 ± 10.1	<0.01	0.04
WHR	0.88 ± 0.08	0.91 ± 0.08	0.95 ± 0.08	<0.01	<0.01
SBP (mmHg)	135.3 ± 19.3	139.5 ± 20.6	144.3 ± 18.5	0.11	0.18
DBP (mmHg)	85.4 ± 10.7	86.3 ± 8.2	89.7 ± 9.1	0.52	0.07
Total cholesterol (mmoll^−1^)	5.3 ± 1.0	5.2 ± 1.1	5.3 ± 1.1	0.63	0.58
HDL-C (mmoll^−1^)	1.3 ± 0.3	1.2 ± 0.3	1.1 ± 0.2	0.12	0.01
Triglycerides (mmoll^−1^)*	1.4 (0.8)	1.7 (1.2)	2.6 (2.1)	0.03	0.04
FPG (mmoll^−1^)	5.0 ± 0.5	5.4 ± 0.6	8.1 ± 2.1	0.02	<0.01
2 h PLG (mmoll^−1^)	5.4 ± 1.2	8.9 ± 1.5	14.9 ± 3.9	<0.01	<0.01
HOMA-IR (arb.)*	1.6 (1.2)	2.5 (1.8)	6.0 (4.9)	<0.01	<0.01
CVD medication					
Antihypertensive	14 (18)	15 (34)	14 (36)	0.01	0.33
Lipid lowering	16 (21)	12 (29)	11 (28)	0.03	0.89

Data are means ± standard deviation (SD) or median with inter quartile range (IQR)* and *n* (%). WC: waist circumference, WHR: waist Hip Ratio, SBP: systolic blood pressure, DBP: diastolic blood pressure, HDL-C: high-density lipoprotein cholesterol subfraction, FPG: fasting plasma glucose, 2-h PLG: 2 hour post-load (75g-oral glucose tolerance test) glucose. HOMA-IR: homeostasis model insulin resistance.

**Table 2:** Mean biomarker concentrations within glucose groups.

	NGT	IGT	T2DM	*P *
Adiponectin (*μ*gmL^−1^)*				
Men	10.6 (6.48)	9.3 (5.0)	6.9 (3.8)	0.07
Women	18.6 (11.8)	15.7 (10.0)	11.0 (4.9)	0.02
Total	13.6 (9.7)	12.4 (7.7)	8.5 (5.1)	0.002
Leptin (ngmL^−1^)*				
Men	10.5 (6.8)	14.3 (13.0)	16.2 (13.5)	0.005
Women	34.2 (29.7)	39.9 (35.7)	43.4 (40.7)	0.07
Total	20.3 (17.6)	26.5 (29.0)	26.7 (27.8)	0.05
TNF-*α* (pgmL^−1^)*				
Men	1.76 (1.4)	1.75 (1.2)	2.14 (2.3)	0.92
Women	1.96 (1.1)	1.65 (1.3)	1.80 (1.2)	0.67
Total	1.84 (1.3)	1.70 (1.3)	2.01 (1.6)	0.77

*Data are median + inter quartile range (IQR).

*P* Nonparametric samples median test.

**Table 3:** Conditional logistic regression of adipokine biomarkers with insulin resistance.

	*R* ^2^	Wald statistic	Beta coefficient (95% CI)	*P *
Model 1				
Adiponectin	0.29	12.41	0.91 (0.85, 0.95)	<0.01
Leptin	0.23	0.09	0.99 (0.97, 1.01)	0.78
TNF-*α*	0.22	0.09	1.03 (0.85, 1.23)	0.03
Model 2				
Adiponectin	0.28	13.13	0.89 (0.85, 0.95)	<0.01
Leptin	0.21	1.69	1.01 (0.99, 1.01)	0.20
TNF-*α*	0.20	0.07	1.00 (1.01, 1.12)	0.93

HOMA-IR > 3.0 used to define insulin resistance for categorical dependent variable.

Model 1: age, gender, BMI, smoking, Cardioprotective medication.

Model 2: age, gender, waist circumference, smoking, total cholesterol.

## References

[1] Scherer PE, Williams S, Fogliano M, Baldini G, Lodish HF (1995). A novel serum protein similar to C1q, produced exclusively in adipocytes. *The Journal of Biological Chemistry*.

[2] Haluzik M, Parizkova J, Haluzik MM (2004). Adiponectin and its role in the obesity-induced insulin resistance and related complications. *Physiology Research*.

[3] Stefan N, Stumvoll M (2002). Adiponectin: its role in metabolism and beyond. *Hormone and Metabolic Research*.

[4] Li S, Shin HJ, Ding EL, van Dam RM (2009). Adiponectin levels and risk of type 2 diabetes. A systematic review and meta-analysis. *The Journal of the American Medical Association*.

[5] Lindsay RS, Funahashi T, Hanson RL (2002). Adiponectin and development of type 2 diabetes in the Pima Indian population. *The Lancet*.

[6] Wannamethee SG, Lowe GDO, Rumley A, Cherry L, Whincup PH, Sattar N (2007). Adipokines and risk of type 2 diabetes in older men. *Diabetes Care*.

[7] Bayés B, Granada ML, Pastor MC (2007). Obesity, adiponectin and inflammation as predictors of new-onset diabetes mellitus after kidney transplantation. *American Journal of Transplantation*.

[8] Snehalatha C, Mukesh B, Simon M, Viswanathan V, Haffner SM, Ramachandran A (2003). Plasma adiponectin is an independent predictor of type 2 diabetes in Asian Indians. *Diabetes Care*.

[9] Snijder MB, Heine RJ, Seidell JC (2006). Associations of adiponectin levels with incident impaired glucose metabolism and type 2 diabetes in older men and women: the Hoorn study. *Diabetes Care*.

[10] Koenig W, Khuseyinova N, Baumert J, Meisinger C, Löwel H (2006). Serum concentrations of adiponectin and risk of type 2 diabetes mellitus and coronary heart disease in apparently healthy middle-aged men: results from the 18-year follow-up of a large cohort from Southern Germany. *Journal of the American College of Cardiology*.

[11] Spranger J, Kroke A, Möhlig M (2003). Adiponectin and protection against type 2 diabetes mellitus. *The Lancet*.

[12] Knobler H, Benderly M, Boyko V (2006). Adiponectin and the development of diabetes in patients with coronary artery disease and impaired fasting glucose. *European Journal of Endocrinology*.

[13] Choi KM, Lee J, Lee KW (2004). Serum adiponectin concentrations predict the developments of type 2 diabetes and the metabolic syndrome in elderly Koreans. *Clinical Endocrinology*.

[14] Daimon M, Oizumi T, Saitoh T (2003). Decreased serum levels of adiponectin are a risk factor for the progression to type 2 diabetes in the Japanese population: the Funagata study. *Diabetes Care*.

[15] Nakashima R, Kamei N, Yamane K, Nakanishi S, Nakashima A, Kohno N (2006). Decreased total and high molecular weight adiponectin are independent risk factors for the development of type 2 diabetes in Japanese-Americans. *Journal of Clinical Endocrinology and Metabolism*.

[16] Rabe K, Lehrke M, Parhofer KG, Broedl UC (2008). Adipokines and insulin resistance. *Molecular Medicine*.

[17] Sattar N, Wannamethee SG, Forouhi NG (2008). Novel biochemical risk factors for type 2 diabetes—pathogenic insights or prediction possibilities?. *Diabetologia*.

[18] Whincup PH, Gilg JA, Papacosta O (2002). Early evidence of ethnic differences in cardiovascular risk: cross-sectional comparison of British South Asian and white children. *British Medical Journal*.

[19] Abate N, Chandalia M, Snell PG, Grundy SM (2004). Adipose tissue metabolites and insulin resistance in non-diabetic Asian Indian men. *Journal of Clinical Endocrinology and Metabolism*.

[20] Valsamakis G, Chetty R, McTernan PG, Al-Daghri NM, Barnett AH, Kumar S (2003). Fasting serum adiponectin concentration is reduced in Indo-Asian subjects and is related to HDL cholesterol. *Diabetes, Obesity and Metabolism*.

[21] Martin M, Palaniappan LP, Kwan AC, Reaven GM, Reaven PD (2008). Ethnic differences in the relationship between adiponectin and insulin sensitivity in South Asian and Caucasian women. *Diabetes Care*.

[22] Raji A, Gerhard-Herman MD, Warren M (2004). Insulin resistance and vascular dysfunction in nondiabetic Asian Indians. *Journal of Clinical Endocrinology and Metabolism*.

[23] Ferris WF, Naran NH, Crowther NJ, Rheeder P, van der Merwe L, Chetty N (2005). The relationship between insulin sensitivity and serum adiponectin levels in three population groups. *Hormone and Metabolic Research*.

[24] Wasim H, Al-Daghri NM, Chetty R, McTernan PG, Barnett AH, Kumar S (2006). Relationship of serum adiponectin and resistin to glucose intolerance and fat topography in South-Asians. *Cardiovascular Diabetology*.

[25] Mente A, Razak F, Blankenberg S (2010). Ethnic variation in adiponectin and leptin levels and their association with adiposity and insulin resistance. *Diabetes Care*.

[26] Luo M, Oza-Frank R, Mohan V, Narayan KM, Gokulakrishnan K (2010). Serum total adiponectin is associated with impaired glucose tolerance in Asian Indian females but not in males. *Journal of Diabetes Science and Technology*.

[27] Bansal N, Anderson SG, Vyas A (2011). Adiponectin and lipid profiles compared with insulins in relation to early growth of British South Asian and European children: the manchester children’s growth and vascular health study. *Journal of Clinical Endocrinology and Metabolism*.

[28] Snehalatha C, Yamuna A, Ramachandran A (2008). Plasma adiponectin does not correlate with insulin resistance and cardiometabolic variables in non-diabetic Asian Indian teenagers. *Diabetes Care*.

[29] Gray LJ, Tringham JR, Davies MJ (2010). Screening for type 2 diabetes in a multiethnic setting using known risk factors to identify those at high risk: a cross-sectional study. *Vascular Health and Risk Management*.

[31] Khunti K, Taub N, Webb DR (2012). Validity of self-assessed waist circumference in a multi-ethnic UK population. *Diabetic Medicine*.

[32] Zhu N, Pankow JS, Ballantyne CM (2010). High-molecular-weight adiponectin and the risk of type 2 diabetes in the ARIC study. *Journal of Clinical Endocrinology and Metabolism*.

[33] Mohan V, Deepa R, Pradeepa R (2005). Association of low adiponectin levels with the metabolic syndrome —the Chennai Urban Rural Epidemiology study (CURES-4). *Metabolism*.

[34] Retnakaran R, Qi Y, Connelly PW, Sermer M, Hanley AJ, Zinman B (2010). Low adiponectin concentration during pregnancy predicts postpartum insulin resistance, beta-cell dysfunction and fasting glycaemia. *Diabetologia*.

[35] Drapeau S, Doucet É, Rabasa-Lhoret R, Brochu M, Prud’homme D, Imbeault P (2011). Improvement in insulin sensitivity by weight loss does not affect hyperinsulinemia-mediated reduction in total and high molecular weight adiponectin: a MONET study. *Applied Physiology, Nutrition and Metabolism*.

[36] Cook JR, Semple RK (2010). Hypoadiponectinemia—cause or consequence of human “insulin resistance”?. *Journal of Clinical Endocrinology and Metabolism*.

[37] Yudkin JS, Yajnik CS, Mohamed-Ali V, Bulmer K (1999). High levels of circulating pro-inflammatory cytokines and leptin in urban, but not rural, Indians. A potential explanation for increased risk of diabetes and coronary heart disease. *Diabetes Care*.

[38] Banerji MA, Faridi N, Atluri R, Chaiken RL, Lebovitz HE (1999). Body composition, visceral fat, leptin, and insulin resistance in Asian Indian men. *Journal of Clinical Endocrinology and Metabolism*.

[39] Hotamisligil GS, Spiegelman BM (1994). Tumor necrosis factor *α*: a key component of the obesity-diabetes link. *Diabetes*.

[40] Wang Y, Lam KSL, Yau MH, Xu A (2008). Post-translational modifications of adiponectin: mechanisms and functional implications. *Biochemical Journal*.

