# Supplementary material for: Adipocytokine Associations with Insulin Resistance in British South Asians
Source: J Diabetes Res. 2013 Feb 25;2013:561016. doi: 10.1155/2013/561016 (PMC3647556; doi:10.1155/2013/561016)
Supplement: Supplementary file 1 — Bivariate associations with HOMA-IR derived insulin resistance for the entire study population are provided as supplementary electronic material. [file 561016.f1.doc]

**Supplementary Electronic Material.**

**Univariate correlation of adiponection, leptin and TNF-α with various biometrics**

|  |  | Age  (yrs) | Gender  (Male) | BMI | WHR | HOMA-IR | SBP | HDL-C |
| --- | --- | --- | --- | --- | --- | --- | --- | --- |
| Adiponectin | r | 0.01 | 0.12 | - 0.24 | - 0.31 | - 0.18 | - 0.09 | - 0.11 |
|  | p | 0.34 | 0.25 | 0.02 | <0.01 | 0.17 | 0.41 | 0.35 |
| Leptin | r | 0.17 | 0.35 | 0.20 | 0.30 | 0.45 | 0.05 | 0.25 |
|  | p | 0.14 | 0.03 | 0.09 | <0.01 | <0.01 | 0.66 | 0.03 |
| TNF-α | r | 0.31 | - 0.18 | - 0.16 | - 0.03 | 0.34 | 0.22 | - 0.07 |
|  | p | 0.08 | 0.11 | 0.17 | 0.83 | <0.01 | 0.04 | 0.54 |
